# A National Pension Fund for Hospital Officials and Nurses

**Published:** 1887-04-30

**Authors:** 


### A National Pension Fund for Hospital Officials and Nurses.

The Views of Officials, Sisters, Nurses, and others.

### By W. H. Hughes, Secretary of the Cancer Hospital, Brompton.

I am very glad to see that the proposal to establish a "National Pension Fund" is likely to be carried out. It is undoubtedly a step in the right direction, and if a provisional committee were formed and a prospectus drawn up for circulation amongst hospital *employés*, setting forth the objects and benefits of the proposed fund, I do not think there would be any lack of applicants for membership. If the matter were put in some such practical form, it would, in my opinion, be more likely to attract the attention of those most interested.

### By W. M. Tollit, Hon. Sec. of the Totnes Cottage Hospital.

I have been asked by the Matron of this hospital to write you concerning the "National Pension Fund" for Hospital Nurses, which is being advocated in your paper. I have not had an opportunity of studying the subject, as I do not see your weekly issue. To assist the maintenance of such an institution, I consider a fund should be established, not only by the hospitals, but by all the nurses in the establishments—the latter contributing a monthly payment, and hospitals in proportion to the number of nurses employed—that is to say, all those who are qualified after a term of probation. I think it not only necessary to provide a pension after a given number of years' service, but to provide a home for the sick and aged, who from other circumstances have not the means to provide for themselves, and that an additional pension should be allowed in proportion to the amount paid and collected by each person during their past years of service. Many persons would willingly subscribe to the fund for any deserving nurse, who intended in after years to participate in the fund; and it would be an inducement to all lady visitors and others connected with small institutions to encourage the nurses in their well-doing, feeling that no provision is made for a superannuation fund in their declining years. I know of no institution that provides for such a case, and it is but few that make provision for sickness and old age, or have from their limited income any power of doing so except by a small donation or subscription. Thus the necessity of all institutions supporting a scheme that with their assistance (enforcing a monthly subscription from all nurses, and soliciting external aid) shall be in a position to afford relief, either by an annuity or asylum, to all deserving members. I have not seen the rules of such an institution, but feel sure, if such a one is put fairly before the general public, no lack of funds will be wanting for its support, provided candidates are only eligible who have received hospital or infirmary training, and served a certain number of years, and can produce a genuine hospital certificate of efficient training. Surely, there will be no difficulty in this Jubilee Year in establishing such an institution in aid of those whose life is spent in administering to the sick and needy, and I hope through your valuable columns it may be an established fact before the end of the present year.

### By Miss Smith, Matron of the Miller Hospital, Greenwich.

I beg to send in my name as a most sincere advocate of the scheme for promoting a National Pension Fund for Nurses; I shall be pleased to subscribe to the same as soon as it is reliably formulated. The want of such a provision against the chances of sickness or old age has long been an anxiety to me, and to many others whom I know. My five nurses also much approve the scheme, and will be glad to avail themselves of the provision for future difficulties as soon as possible. I send you the names of my five nurses, as well as my own.

### By One Who Would Gladly Join.

"An Earnest Worker" says truly—nurses are an "underpaid" class, considering the long hours, the short time off duty, the hard, dirty (often disgusting) work they have to do. There is no romance about hospital work, notwithstanding the romantic verbiage sometimes written and talked about it. It is as we say—real hard work; and domestic servants, tradesmen's daughters, to say nothing of ladies who take up nursing as probationers, have never worked so hard before. Numbers of nurses have small chance of "saving"—there are so many at home to be helped; but at the same time we find it most difficult to persuade nurses to "put away" every quarter, so much money goes in little things; and young people *cannot* and *will not* see the necessity for providing for the "rainy day" which sooner or later comes to all. "The National Pension Fund" is a good idea, but nurses like to feel that, should they die, some relation would be able to draw the money, and so reap the benefit. Would that be so? May we add a word for the matrons and sisters who are most of them "reduced" (at least, when *so many* must work for a living, it cannot be just to fill up situations with those who have *no need of pay*). Many have from £20 a year of their own, or may at some time expect it; but that would hardly keep body and soul together, if rent has to be paid. It would, however, go a long way if only used for food and clothes. It would cheer many a one to know that up and down our land there were "Nurses' Alms-houses," where they could call two or three rooms *their own*. Will no one start the idea for our hard-worked sisters and matrons, in this year of Jubilee?

### By a Cottage Hospital Nurse.

I have been looking very anxiously for further particulars of the Nurses' Pension Fund, as, if the regulations are such as I can conform to, I shall be glad to subscribe annually, and will also, if necessary, give a donation to help in starting it.

Any hospital officials or nurses willing to co-operate in the establishment of this fund, should promptly send their name and address to Mr. Howard J. Collins, Secretary, The Hospitals Association, Norfolk House, Norfolk Street, W.C.

